# Facility newborn and stillbirth data use and enabling factors at different levels of the health system: findings of the IMPULSE study across 142 sites in the Central African Republic, Ethiopia, Tanzania, and Uganda

**DOI:** 10.7189/jogh.15.04295

**Published:** 2025-12-19

**Authors:** Firehiwot Abathun, Paolo Dalena, Rornald Muhumuza Kananura, Jacqueline Minja, Ousman Mouhamadou, Mary Ayele, Louise Tina Day, Francesca Tognon, Lorenzo Giovanni Cora, Ilaria Mariani, Sara Geremia, Giovanni Putoto, Joy Elizabeth Lawn, Tamirat Awel, Felix Bundala, Donat Shamba, Peter Waiswa, Marzia Lazzerini

**Affiliations:** 1 Doctors with Africa CUAMM, Addis Ababa, Ethiopia; 2 Institute for Maternal and Child Health IRCCS Burlo Garofolo, Trieste, Italy; 3 University of Trieste, Trieste, Italy; 4 Demographic Dynamic and Population Health Unit, African Population and Health Research Center, Dakar, Senegal; 5 Ifakara Health Institute, Ifakara, Tanzania; 6 Doctors with Africa CUAMM, Bangui, Central African Republic; 7 London School of Hygiene & Tropical Medicine, London, UK; 8 Doctors with Africa CUAMM, Padua, Italy; 9 Institute for Maternal and Child Health IRCCS Burlo Garofolo, WHO Collaborating Centre, Trieste, Italy; 10 Federal Ministry of Health: Strategic Affairs Executive Office, Addis Ababa, Ethiopia; 11 Ministry of Health, Dodoma, Tanzania; 12 Department of Health Policy Planning and Management, Makerere University of Public Health, Kampala, Uganda; * Joint senior authorship.

## Abstract

**Background:**

Improving data quality and use is a priority identified by the World Health Organization (WHO) to reduce stillbirths and newborn deaths; however, few studies have documented newborn and stillbirth data use in the African Region. To address this gap, we conducted a cross-sectional study from November 2022 to July 2024 in 12 regions and four city administrations across the Central African Republic (CAR), Ethiopia, Tanzania, and Uganda.

**Methods:**

We collected data using Every Newborn – Measurement Improvement for Newborn & Stillbirth Indicators (EN-MINI) tools, through direct observation, and from routine electronic and/or paper-based forms and reports. We analysed both the overall and country-level samples following the Performance of Routine Information System Management User’s Kit.

**Results:**

We assessed 142 sites, comprising 93 health facilities and 49 data offices. Enabling factors, such as electronic systems, guidelines, annual plans, and feedback mechanisms, were highly available in Ethiopia (n/N = 22/30), moderately available in Uganda and Tanzania (n/N = 15/30 and n/N = 12/30), and scarce in the CAR (n/N = 1/30), with key indicators in all countries being ≥80%. Key data-use indicators showed similar patterns across countries, but lower frequencies (Ethiopia: n/N = 15/66; Uganda: n/N = 14/66; Tanzania: n/N = 5/66; the CAR: n/N = 1/66). Decisions documented in meetings rarely focussed on healthcare quality improvement, particularly at the district level (47.1% in Tanzania *vs.* 44.4% in Uganda *vs.* 30% in Ethiopia *vs.* 0% in the CAR). Data dissemination to public representatives was also subpar (40.8% in facilities, 71.4% in subnational offices). Among 141 end-users, 100% of respondents in the CAR, 75–82.4% in Tanzania and Uganda expressed the need to improve the newborn and stillbirth data use compared to 16.7–21.7% in Ethiopia.

**Conclusions:**

Newborn and stillbirth data use was low, despite variations across countries and health system levels. Evidence-based decision-making in health service delivery remains a priority action to reduce stillbirths and newborn deaths. This study identified context-specific, sustainable, and scalable interventions co-created with end-users to ensure wider newborn and stillbirth data use.

According to the latest estimates of 2022, 2.3 million newborns died globally, accounting for 47% of all child deaths under the age of five [1]. Sub-Saharan Africa recorded one of the highest neonatal mortality rates in 2022, at 27.1 deaths per 1000 live births and 21.1 stillbirths per 1000 total births [1–4]. The majority of neonatal deaths occur during the first week of life and are predominantly caused by preventable causes, which could be reduced through improvements in the related quality of care [2].

All countries aim to reduce neonatal mortality to at least 12 deaths per 1000 live births and 12 or fewer stillbirths per 1000 total births by 2030 – the threshold set in the 2030 Sustainable Development Goals (SDGs) [5]. According to a recent World Health Organization (WHO) progress report, Ethiopia and Tanzania are among the top 10 countries with the highest number of neonatal and maternal deaths, while the Central African Republic (CAR) is recognised as a fragile state carrying a significant burden of preventable maternal and newborn deaths [5]. Current prevalence rates of neonatal deaths in several low-income countries, including the CAR, Ethiopia, Tanzania and Uganda (**Table 1**), show that substantial efforts are still needed to progress and meet the 2030 SDGs [5]. Since between 52.2% and 73.4% [6] of all births in the CAR, Ethiopia, Tanzania, and Uganda occur in healthcare facilities, improving the quality of routine health information systems (RHISs) is vital for reducing stillbirths and newborn deaths.

**Table 1:** IMPULSE study: mortality rates of countries selected for the IMPULSE Phase 1 study

Mortality indicators	CAR	Ethiopia	Tanzania	Uganda
Number of neonatal deaths per 1000 live births	32	27	20	18
Number of stillbirths per 1000 total births	26	21	18	15
Institutional births, %	52.2	47.5	62.6	73.4

CAR – Central African Republic

Improving data quality and use as a core component for the provision of quality maternal and newborn care has been identified as a key action for reducing stillbirths and newborn deaths [5]. High-quality data are needed for enabling measurement, programme tracking and planning, thereby promoting accountability and action toward progress [5]. Health facility data specifically enable healthcare managers to identify local priorities for quality improvement, determine resource needs, guide medical supply and equipment purchase, and plan any activity, such as community outreach activities [7,8].

However, due to the absence of stillbirth registration policies, many countries have not fully prioritised data to inform action and reduce stillbirths [5]. Even more concerning, previous studies suggested that data use for activity planning in low-middle-income countries (LMICs) remains low [7,9,10], and the high data collection burden was not matched with effective data use for decision-making [11]. In Ethiopia, studies have highlighted that the utilisation of health information from the health management information system (HMIS) was low, with routine information usage at public health institutions being lower than regional and national expectations [12,13]. Similarly, studies in Ethiopia and Tanzania suggested that data are mostly collected for reporting purposes in health settings, with minimal utilisation of the information to inform programmatic decision-making [10,14].

There is a lack of multi-country studies in sub-Saharan Africa reporting a comprehensive assessment of newborn and stillbirth data use and related enabling factors at different levels of the health system. While studies have reported on factors such as the availability of existing tools and technologies, capacity strengthening, and governance [15–17], none have explored multiple other enabling factors, including the existence of related data use guidelines, annual plans defining targets, relevant feedback systems, performance monitoring team (PMT) meetings, and actual data processing, critical for enabling data use. A better understanding of current newborn and stillbirth data use and related enabling factors at the facility, subnational, and national levels may help identify key gaps and opportunities for improving the quality of care and, therefore, newborn survival.

IMPULSE is a collaborative project among various institutions – the London School of Hygiene and Tropical Medicine (LSHTM), the Ifakara Health Institute in Tanzania, Makerere University in Uganda, the WHO Collaborating Centre for Maternal and Child Health Italy, and Doctors with Africa *Collegio Universitario Aspiranti Medici Missionari* (CUAMM), which is an Italian NGO working in Africa. This paper is one of several studies reporting on the findings of the IMPULSE phase 1 study in the CAR, Ethiopia, Tanzania, and Uganda related to facility newborn data quality, use, processes and input factors. This research utilised the Performance of Routine Information System Management (PRISM) [18] framework (Figure S1 in the **Online Supplementary Document****)**, which provides a conceptual model of RHIS performance elaborating the various determinants at input, processes, output and outcome levels of RHIS to provide a structured analysis of many factors contributing to data quality and use.

The IMPULSE project focussed on newborn and stillbirth data use and key enabling factors in 142 sites (93 facilities, 49 district health offices) across four countries. In addition to data use, it explored, in line with the PRISM framework, data quality and various technical, organisational, and behavioural input factors affecting data processing and utilisation. These additional findings are reported in separate papers [19-22].

## METHODS

### Study design and participants

We conducted a cross-sectional study across 12 regions and four city administrations in the CAR, Ethiopia, Tanzania, and Uganda, and reported our findings in accordance with the STROBE guidelines [23] (Figure S1, Table S1, and Table S2 in the **Online Supplementary Document**). We selected regions by the following three criteria:

1. heterogeneity, *i.e.* regions with different characteristics, including underperforming for maternal and neonatal mortality, hard-to-reach areas, or humanitarian settings;
2. regions where the implementing agency, Doctors with Africa CUAMM , had an office/ project, or nearby regions, that could facilitate coordination
3. regions prioritised by ministries of health (MoHs) as a national priority.

We defined a sample size using the PRISM lot quality assurance sampling (LQAS) methodology, requiring a minimum of 19 facilities in each country.

We focussed on facilities providing comprehensive newborn essential care (*i.e.* only comprehensive emergency obstetric care health facilities (CEmOC)), with or without neonatal inpatient care, and selected a pre-defined number, stratified by level (from national hospitals to health centres) and type (urban, rural, public, private, and faith-based), and their linked data offices at national, and subnational (regional and district) levels. We pre-defined a fixed number of facilities in each category for each region (Table S3 in the **Online Supplementary Document**), selecting higher-level facilities with a higher number of deliveries, and, for practical reasons, opting for health centres located in the same district as these larger facilities. We also assessed one national hospital or the largest facility in each country.

We excluded any sites in geographical areas with active conflicts or where the road conditions prevented safe access (Ethiopia: five health facilities and one subnational office; Uganda: one health facility). In the CAR, we focussed the assessment in Bangui city and health regions 1, 2, and 7 due to internal security constraints, but included seven basic emergency obstetric and neonatal care health facilities to increase the sample size.

### Data collection tools

We collected data between November 2022 and July 2024 using the Every Newborn – Measurement Improvement for Newborn & Stillbirth Indicators (EN-MINI)-PRISM tools version 2, specifically, tools 2a and 2b. We co-created this set of open-access tools as an adaptation of the PRISM tools, which have been used for over 20 years [18]. We updated version 2 of the EN-MINI tools and pilot-tested them with additional research questions from the IMPULSE study.

Since the EN-MINI tools were originally released in English and Swahili, our study coordinators translated them into Amharic (for use in Ethiopia) and French (for use in the CAR) before the start of data collection. We directly observed and/or extracted most data from existing documents (*e.g.* guidelines, reports, meeting minutes). The additional research questions in the EN-MINI version 2 included end-users’ perspectives regarding RHIS, collected through interviews with key staff [24]. We defined key staff in the EN-MINI tool instructions for data offices, those working in the HMIS unit, the plan and policy unit, the quality unit, the maternal and child health program staff, the regional health bureaus or the central MoH; for health facilities, labour and delivery ward or neonatal intensive care unit coordinators, HMIS officers, plan and policy unit representatives.

### Data quality assurance

We collected data directly on a SurveyCTO (Dobility, Inc., Washington, D.C., USA) digital platform, which included validations for completeness and plausibility. On each site, a team of three to six collectors collected data in respondents’ language of choice (Amharic, English, French, Swahili), under the supervision of the IMPULSE study coordinators (FA, MA, JM, MKR, OM). Data collectors and study coordinators underwent formal training, field practice, and a series of preliminary meetings to clarify any doubts; received a file where all questions and answers were recorded and made available; and were introduced into a WhatsApp group to solve any upcoming new questions in real-time.

During data collection, study coordinators conducted regular monitoring and evaluation daily, where they utilised a pre-defined, field-tested Excel workbook to review data timeliness, completeness, and sample size collected daily by a supervisor (FT). Missing or implausible data were discussed in real-time. To further check data completeness, internal and external consistency, and plausibility, independent data analysts (IM, SG, PD) conducted four rounds of interim analyses upon the start of data collection. Study coordinator and supervisors (MA, FA, JM, MKR, OM) provided reports of these analyses; we discussed interim results and updated and optimised data collection accordingly. In the data analysis phase, we cross-checked our results and automated tools' results, which were included in EN-MINI-PRISM tools and cover several of the existing variables. Additionally, we held data validation workshops in each country to discuss IMPULSE Phase 1 study findings and their implications.

### Data analysis

We analysed all variables descriptively, *i.e.* calculating absolute and percentage frequencies based on the PRISM User’s Kit [18]. Accordingly, we calculated two combined indicators, which utilise a weighted average percentage, known as the average score, ranging from 0 to 100. We also calculated how many indicators for each country, for each domain, reached a frequency ≥80%.

We analysed all data on the overall sample, by country, and by level (facility and office). We also performed additional descriptive analyses beyond the PRISM User’s Kit. We tested differences in characteristics by groups with a χ^2^ or a Fisher exact test, as appropriate. We did not consider subgroup analyses by region as appropriate, due to the low sample size in each region and the large number of sub-questions in the EN-MINI tools (*e.g.* questions to be answered only for those answering ‛Yes’ on a previous question), resulting in many variables with a low sample size when further subgrouped by regions. Multivariate analyses exploring how individual site characteristics associated with key outcomes of data use were not feasible using existing PRISM composite indicators, because, according to the PRISM methodology, these are calculated as aggregate variables at the country level (not at each site).

We performed statistical analyses using *R*, version 4.2.1 (R Core Team, Vienna, Austria). A *P*-value of <0.05 was considered statistically significant; all the tests were two-tailed.

### Ethical aspects

We collected data according to the General Data Protection Regulations. We ensured anonymity in the survey by not collecting any information that could disclose participants’ identities. Moreover, we collected only aggregate routine health facility data from each facility/office. For staff interviews, we informed the participants about the study’s objectives and methods, including their rights to decline participation. We also provided each participant with an information sheet and obtained their written consent before they responded to the survey. We used password-protected tablets or phones following EN-MINI-PRISM version 2 open data kit forms for data transmission and storage, which we uploaded onto encrypted servers hosted by Survey CTO through a community license. We collected region and site name and key informant role, but no individual identifiers. We downloaded data by country and ensured they did not include any key informant identifiers before securely transferring it to the study group. We stored paper documents in locked filing cabinets.

## RESULTS

### Characteristics of the sample

The overall sample for the IMPULSE study included 154 sites, comprising 57 subnational offices and 97 facilities. The sample size varied based on the data collection tool utilised. Here, we included a total of 142 sites, comprising 93 healthcare facilities and 49 corresponding subnational data offices, where we conducted assessments with EN-MINI-PRISM tools 2a and 2b (Table S4 in the **Online Supplementary Document**). Twenty (14.1%) were located in the CAR (14 facilities and 6 related district health offices), 30 (21.1%) in Ethiopia (24 facilities and 6 district health offices), 44 (30.9%) in Tanzania (27 facilities and 17 district health offices, including 3 regional health bureaus), and 48 (31.2%) in Uganda (28 facilities and 20 district health offices).

Overall, 71 (76.3%) of the facilities were government-owned, and 22 (23.7%) were either private for-profit or private not-for-profit; 61 (65.6%) facilities were located in urban settings, while 32 (34.4%) were in rural settings. Among facilities, 15 (16.2%) were either national or third level of referral hospitals, 39 (41.9%) facilities were district provincial hospitals (second level of referral) and 39 (41.9%) were health centres (first level of referral) providing CEmOC services except 7 basic emergency obstetric care (BEmOC) facilities in the CAR, given the lack of CEmOC facilities in the country) (Table S4 in the **Online Supplementary Document**).

### Data processing and use

**Figure 1:**
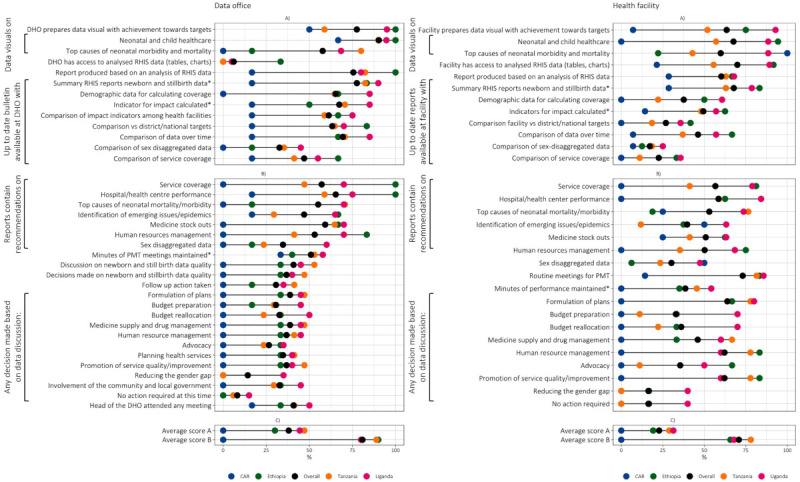
IMPULSE study: data processing and use at the district health office and facility levels (93 facilities, 49 health offices). **Panel A.** Data processing. **Panel B.** Data use for quality improvement. **Panel C.** PRISM composite scores. Each dot represents the value for one country. When two values overlap, one dot may not be visible. PRISM composite scores were calculated according to the PRISM manual for analysis, called PRISM User’s Kit [18]. Average score A: average score on the use of routine data for RHIS quality improvement, performance review, and evidence-based decision making. Average Score B: average score on the use of routine data for RHIS quality improvement, performance review, and evidence-based decision making (among facilities maintaining performance monitoring/management meeting minutes for the three review months. CAR – Central African Republic, DHO – district health office, MoH – Ministry of Health, PMT – Performance management team, RHIS – routine health information system.

We observed a pattern of distribution of frequencies for data processing and use across countries, with the CAR showing the lowest frequencies and Ethiopia the highest, followed by Tanzania or Uganda, depending on the specific variable (**Figure 1**).

### Data visual presentation

All subnational data offices in Ethiopia and 75% of facilities used data to prepare visuals showing progress towards targets (**Figure 1**, Panel A). This compares with 95% and 92.9% in Uganda, 58.8% and 51.9% in Tanzania, and 50% and 7.1% in the CAR, respectively (*P*-value for comparison among facilities <0.001, *P*-value for comparison among data offices = 0.01). However, data visuals on the leading causes of neonatal mortality and morbidity were limited in Ethiopia, both at the data office (16.7%) and at the facility level (22.2%).

### Data reporting

The availability of a narrative analytical report the highest in Ethiopia (100% and 66.7%, at data office and facility level, respectively), followed by Uganda (80% and 67.9%) and Tanzania (82.4% and 63%), and the lowest in the CAR (16.7% and 28.6%) (*P*-value for comparison among facilities = 0.007, *P*-value for comparison among data offices = 0.018).

The contents of narrative analytical reports were heterogeneous. The key common gap across the four countries was between the facility and the district or national targets (26.9% on all facilities, ranging from 0% in the CAR to 41.7% in Ethiopia; *P* = 0.019 across the four countries). Among subnational offices, common gaps included the comparison of service coverage (46.9% overall, range 16.7–66.7%); and comparisons of sex-disaggregated data (32.7% overall, range 0–45%) (**Figure 1****,** Panel A; Tables S5 and S6 in the **Online Supplementary Document**).

### Data use

Regarding data use for quality improvement (**Figure 1**, Panel B; Tables S5 and S6 in the **Online Supplementary Document**), we found that reports based on analysed data at facility level that included written recommendations varied, with recommendations on facility performance being the most frequent both at facility level (58.5% overall, ranging from 0% in the CAR to 84.2% in Uganda; *P* < 0.001 across the four countries) and data office level (65.3% overall, ranging from 16.7% in the CAR to 100% in Ethiopia; *P* = 0.014 across the four countries). Conversely, recommendations on the leading causes of neonatal mortality were available in approximately half of the facilities (53.0% overall, ranging from 25.0% in the CAR to 76.5% in Tanzania; *P* < 0.001), as well as in data offices (55.1% overall, ranging from 18.8% in the Ethiopia to 70.6% in Tanzania; *P* = 0.002 across the four countries).

Performance monitoring and management team meetings were consistently documented in the sites of Ethiopia, Tanzania, and Uganda, both at facilities (81.5–85.7%) and data offices, and only in a minority of sites in the CAR (14.3% at facilities, 50% of data offices; *P* < 0.001). At the facility level, teams met once (22.9%) to three times (49.9%) in the last three months, while at the data office level, meetings happened once in the last three months (57.5%) or not at all (17.1%). Moreover, both facilities (0% in the CAR, 35% in Ethiopia, 45.5% Tanzania, 54.2% Uganda*; P =* .006) and data offices (33.3% in the CAR, 40% in Ethiopia, 52.9% Tanzania, 57.8% Uganda; *P* = 0.696) poorly maintained meeting minutes.

Decisions documented in these minutes rarely focussed on improving the quality of healthcare services (**Figure 1**, Panel B; Tables S5 and S6 in the **Online Supplementary Document**), in particular at the data office level (47.1% in Tanzania, 40% in Uganda, 33.3% in Ethiopia, 0% in the CAR).

We calculated the number of key data use indicators reaching frequency of ≥80% and found that Ethiopia had the highest range of items at data office and at the facility level (n/N = 8/36 and 7/30, respectively; total = 15/66), while the CAR had the lowest range at both levels (n/N = 1/36 and 0/30, respectively; total = 1/66), with Uganda (n/N = 7/36 and 7/30, respectively; total = 14/70) and Tanzania (n/N = 4/36 and 1/30, respectively; total = 5/66) scoring between the two countries at both levels.

### Composite PRISM indexes

The average composite PRISM score on the use of routine data for RHIS quality improvement, performance review, and evidence-based decision making was low at both facility and data office levels in all countries, ranging from zero in the CAR to 31.4% in Uganda for facilities, and from 0% in the CAR to 47.1% in Tanzania for data offices (**Figure 1**, Panel C; Table S7 in the **Online Supplementary Document**). When focussing only on the subsample of facilities and data offices maintaining performance monitoring and management meeting minutes over the previous three months, the score increased substantially at the data office level (Ethiopia 90.0%, Tanzania 88.9%, Uganda 80.0%); but did not reach 80% at the facility level in any country and remained 0% in the CAR.

### Data dissemination outside the health sector

**Figure 2:**
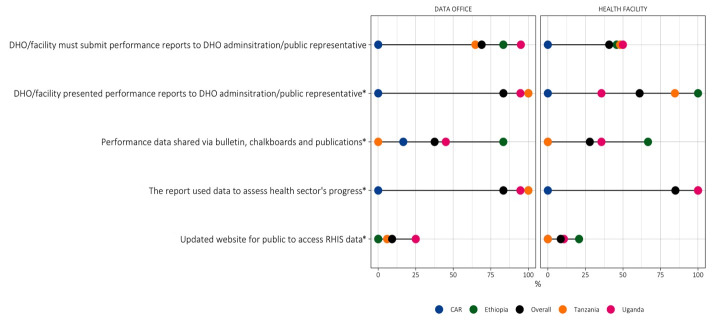
IMPULSE study: data dissemination to the general public (n = 93 facilities, 49 subnational offices). *Observed. In the image, each dot represents the value for one country. When two values overlap, one dot may not be visible. CAR – Central African Republic, DHO – district health office, RHIS – routine health information system.

Data dissemination to the general public largely varied across countries **(****Figure 2**; Table S8 in the **Online Supplementary Document****)**. About half of the facilities in Ethiopia, Tanzania, and Uganda (45.8%, 48.1%, and 50%, respectively) and over two-thirds of data offices (83.3%, 64.7%, and 95%, respectively) were required to submit performance reports on newborn care to councils of public representatives and to district councils, respectively, compared to none in the CAR (*P*-value for comparisons among facilities = 0.009). All assessed facilities in Ethiopia presented reports to public representatives, compared to 84.6% of facilities in Tanzania, 35.7% in Uganda, and 0% in the CAR (*P* < 0.001). Similarly, all data offices in Ethiopia and Tanzania, and 94.7% of those in Uganda, presented data to the local administration, compared to 0% in the CAR (*P* < 0.001). Facilities and data offices rarely relied on updated websites for data dissemination among the public (8.6% at the facility and 12.2% at the office level overall). Instead, they most often shared data through bulletins, chalkboards, and publications (27.9% at the facility and 30.6% at the office level overall), and in other ways.

When the number of key data dissemination indicators reached a frequency of ≥80%, we summarised data at the data office and facility level. The obtained values were the highest in Ethiopia (n/N = 6/10) and the lowest in the CAR (n/N = 0/10), with Uganda (n/N = 4/10) and Tanzania (n/N = 4/10) scoring in between the two countries.

### Enabling factors

**Figure 3:**
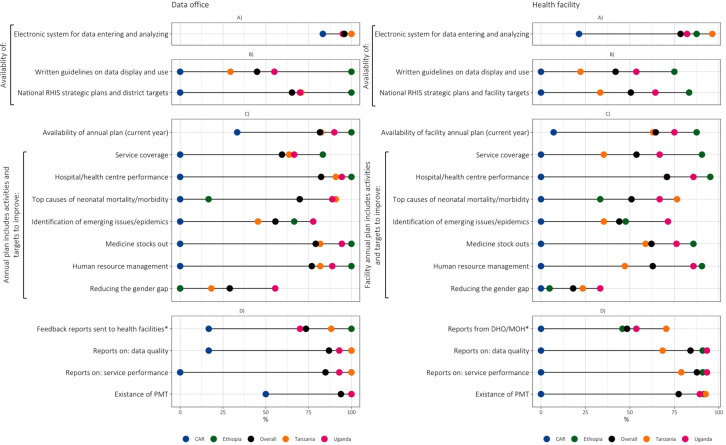
IMPULSE study: enabling factors at data office and facility levels (n = 93 facilities, 49 data offices). **Panel A.** Electronic systems. **Panel B.** Guidelines. **Panel C.** Annual plans. **Panel D.** Feedback systems. *In the last 3 months. Each dot represents the value for one country. When two values overlap, one dot may not be visible. DHO – district health office, MoH – Ministry of Health, PMT – performance monitoring/management team, RHIS – routine health information system.

The availability of functional electronic systems for data entry and analysis was higher in data offices (95.9% overall; 100% in Ethiopia and Tanzania, 95% in Uganda, 83.3% in the CAR) than in facilities (78.5% overall). Notably, in Ethiopia, Tanzania, and Uganda, over 75% of facilities used electronic systems, while in the CAR, only about one in five (21.4%) facilities utilised an electronic system **(****Figure 3**, Panel A; Tables S9 and S10 in the **Online Supplementary Document**). All data offices used District Health Information Software 2 (DHIS2) as a platform for key data analysis.

The availability of relevant documents, such as guidelines for data use, annual plans, and feedback reports (**Figure 3****,** Panels B–D) varied widely across countries, with a similar pattern of distribution, *i.e.* none or very low availability in the CAR and high availability for most items in Ethiopia, and with Uganda and Tanzania scoring between the two countries (**Figure 3**). Specifically, written guidelines on data display and use were available at the facility and data office level in no site in the CAR, 22.2% and 29.4% of sites in Tanzania, 53.6% and 55% in Uganda, and 75% and 100% in Ethiopia (*P*-values for comparison among facilities and data offices <0.001). Similarly, national RHIS strategic plans were missing in the CAR either at the facility and data office level, and were available in 33.3% *vs.* 64.3% *vs.* 83.3% of facilities in Tanzania, Uganda, and Ethiopia, respectively (*P* < 0.001), and in 70% *vs.* 70.6% *vs.* 100% of data offices in Uganda, Tanzania, Ethiopia, respectively (*P* = 0.009).

Likewise, annual plans were available in only one facility in the CAR, compared to 63.0% *vs.* 75.0% *vs.* 87.5% of facilities in Tanzania, Uganda, and Ethiopia, respectively (*P* < 0.001). At data office level, availability was 33% *vs.* 82.4% *vs.* 90% *vs.* 100% in the CAR, Tanzania, Uganda, and Ethiopia respectively (*P* < 0.001). Activities included in annual plans to improve newborn care were primarily related to facility performance at both the facility (70.9%) and subnational data office levels (82.3%). However, there were some specific gaps in each country. In Ethiopia, activities to improve neonatal mortality and morbidity were included in annual plans in 16.6% of data offices and 33.3% of facilities. In Tanzania, activities related to the identification of emerging epidemics were mentioned in 45.4% of annual plans at the data office level. Activities related to reducing gender disparity were globally poorly available in annual plans (ranging at the facility level from 0% in the CAR to 33.3% in Uganda, and at data offices from 0% to 55.5% in the same countries).

A feedback report (from district health offices or central MoH in the last three months) on newborn data quality was available in 0–16.7% of facilities and data offices in the CAR, respectively, compared to 68.4–100% in Tanzania, 93.9–92.3% in Uganda, and 90.9–100% in Ethiopia (*P*-value for comparison among and data offices <0.001). A performance monitoring and management team has not been established in any visited facility in the CAR, but was present in half of the data offices; conversely, most sites in Ethiopia (91.6–100%), Tanzania (92.6–100%) and Uganda (89.3–100%) had such teams.

Our calculation of key enabling factor indicators reaching a frequency of ≥80% at data office and at facility level, showed that Ethiopia had the highest item values (n/N = 12/15 and n/N = 10/15, respectively, total = 22/30), followed by Uganda (n/N = 9/15 and n/N = 6/15, respectively, total = 15/30) and Tanzania (n/N = 10/15 and n/N = 2/15, respectively, total = 12/30), while the CAR had the lowest (n/N = 1/15 items and n/N = 0/15, respectively, total = 1/30) (Table S9 and S10 in the **Online Supplementary Document**).

### End users’ perspective on RHIS improvement

Among 141 end-users interviewed (one per site, with one facility missing), all respondents (100%) in the CAR, and about three-quarters of responders from Uganda (75% at both facility and data office level) and Tanzania (77.8–82.4%) expressed the need for improvement of newborn and stillbirth data use at both facility and subnational levels. This was significantly different from findings in Ethiopia, with only one out of five responders at both the facility and data office level (16.7–21.7%) expressing this need (Figure S3 and Table S11 in the **Online Supplementary Document**).

## DISCUSSION

Here, we presented new evidence on newborn and stillbirth data use across four African countries. According to the IMPULSE findings, Ethiopia, Tanzania, and Uganda consistently performed better than the CAR on data use and related enabling factors. However, upon reviewing routine reports, bulletins, and meeting minutes, we found several specific gaps in newborn and stillbirth indicator use across all four countries, with heterogeneity findings across different indicators. We highlighted variations among various levels in the health system, with facilities generally showing lower rates of analysed newborn data, and subnational (district and regional) data offices showing lower rates of databased decision-making on newborn care during PMT meetings. This included discussions and decisions on newborn and stillbirth data quality. Notably, while we observed enabling factors for data use, the actual utilisation of the data was much lower in all four countries, especially in Ethiopia. The use of sex-disaggregated data on newborns and stillbirth in the decision-making processes was notably low across all countries. Similarly, plans to reduce gender gaps were minimal.

Our findings align, to a large extent, with existing literature. Recent studies in Ethiopia and Tanzania indicated databased decision-making was low, with most of the data processing, analysis, report production and data utilisation for annual planning practised at higher levels of the healthcare system [10,25]. A previous study in Northwest Ethiopia, not specific to newborn data, showed health information utilisation at 51.3% of primary healthcare units, 42.1% of health posts, and the least 38.5% at hospitals [26]. However, other studies from Tanzania, South Africa, and Ethiopia, when compared to findings in our study, reported a slightly higher percentage of facilities holding regular PMT meetings [9,10], using data to assess performance in terms of service coverage [10], RHIS data use for advocacy [9] and data utilisation for resource allocation and planning [12,27]. As shown by other IMPULSE publications, results can vary even in the same country.

Our findings indicated that the CAR had fewer enabling factors that facilitated data analysis, and a consistently lower performance compared to Ethiopia, Tanzania, and Uganda. These findings are further supported by a previous study that identified the CAR as a fragile humanitarian setting and highlighted the issue of unreliable data due to multiple barriers [28].

Concerning the determinants of low data use, a systematic review [29] on data use challenges, exploring technical, organisational, and behavioural determinants, suggested that challenges for data utilisation were more related to organisational and behavioural factors than to technical factors. Other IMPULSE publications are focussing on technical, organisational, and behavioural factors in greater detail [19–22]. One of these assessments investigated organisational factors and noted low budget availability for RHIS activities and gaps in supervision at the facility level. Moreover, training for healthcare workers performing RHIS tasks varied by country and cadre, with many staff lacking training in the last three years, despite the existence of multiple electronic and paper-based recording and reporting tools requiring training [19]. The interoperability of different information systems was also found to be low [19]. A recent qualitative systematic review reported the lack of interoperability among existing tools, and this, along with multiple parallel systems, increased healthcare workers' workload, data duplication, and data collection and reporting to fulfil a requirement [30].

End users’ perspectives showed the need for improvement in RHIS in all countries, with reported lower frequencies in Ethiopia. Previous qualitative studies in Ethiopia reported that the lack of strong leadership, poor infrastructure, multiple data collection and reporting tools, shortage of human resources, lack of recognition and incentives, and capacity building were barriers to improving data quality and use [31,32]. Our findings may be influenced by multiple factors, including Ethiopia’s better performance, subjective perception, or cultural factors. In-depth qualitative responses from end users in the IMPULSE study will be presented in other publications for further insight into data use practices and barriers.

A recent systematic review [7] on priority interventions to improve data use showed that the transformation from data collection to data use should include key features such as measures important to neonatal team members, co-development with local providers, immediate access to data for review, and multidisciplinary team involvement. Different approaches have been used in previous research to improve data use, with key ones being: investment in facility-level data collection [7]; regular workshops on data use to improve the understanding of programme managers to think in terms of indicators [32]; data collection forms simplifying and integrating multiple databases into one, such as DHIS2 [24,33,34]; clear leadership and governance systems, communicated to all health workers [25]. The importance of training, mentorship and supervision has been highlighted in multiple studies [10,13,25,26,29,32,35]. One study reported that the majority of health workers believed they had high self-efficacy in data analysis, interpretation and use; however, another study found that only about two-thirds of health workers demonstrated high competency in these skills [13]. Tools and models that provide essential information and facilitate capacity-building were also highlighted in a scoping review [15] as additional interventions for improving data use. Other relevant interventions mentioned in the literature include technical aspects, such as improving the electronic system for transmitting data reports and communications across different levels in the health system [25].

### Limitations and strengths

We acknowledge several limitations of this study. The findings are not directly generalisable outside the study settings; however, the study could be easily replicated in other contexts. Moreover, we found several common gaps across the four countries, and, together with other studies [11,12,19–22,25], these suggest that data use may be substandard in many settings. Furthermore, we found a limited sample size in the CAR (facility and subnational offices) and Ethiopia (subnational offices) due to security reasons (ongoing conflicts). Our decision to include BEmOC facilities may contribute to the underperformance of the CAR in enabling factors, data processing, and data utilisation, since BEmOC facilities differ in infrastructure and resources compared with CEmOC facilities. Furthermore, selecting study sites based on practicality, security, links to the implementing agency, and MoH requests introduces selection bias, representing an additional limitation that affects generalizability. The IMPULSE project will provide each country with detailed findings to enable tailored action at each study site.

The strength of this study is that it provides one of the few comprehensive assessments of newborn and stillbirth data use and its enabling factors in facilities and subnational offices across four African countries, according to a predefined PRISM framework [18]. We utilised systematic methods of data collection, following a pre-defined methodology. We collected most of the data through direct observation. Lastly, we provide data for action, *i.e.* detailed information on newborn and stillbirth data use, which can be immediately utilised to inform policies at both a national and global level, in the light of decreasing stillbirth and improving newborn health and survival.

## CONCLUSIONS

This study calls for action and requires setting-specific tailored interventions to improve the processing and use of newborn and stillbirth data to improve the quality of care, and therefore newborn health and survival. Our findings can serve as a baseline assessment for decision-makers in newborn health programs, providing a foundation for further monitoring of newborn and stillbirth data utilisation in their respective regions, districts, and facilities.

## Additional material


Online Supplementary Document

## References

[1] World Health Organization. Newborn mortality. 14 March 2024. Available: https://www.who.int/news-room/fact-sheets/detail/newborn-mortality. Accessed: 21 June 2024.

[2] Healthy Newborn Network. Save the Children. Data - Healthy Newborn Network. 2024. Available: https://healthynewbornnetwork.org/data/#_when. Accessed: 21 June 2024.

[3] United Nations Inter-agency Group for Child Mortality Estimation, United Nations Children’s Fund. Levels & Trends in Child Mortality Report. New York, USA: United Nations Children’s Fund; 2023. Available: https://childmortality.org/wp-content/uploads/2024/03/UNIGME-2023-Child-Mortality-Report.pdf. Accessed: 8 October 2024.

[4] Arora A. Never Forgotten: The situation of stillbirth around the globe. 9 January 2023. Available: https://data.unicef.org/resources/never-forgotten-stillbirth-estimates-report/. Accessed: 8 October 2024.

[5] World Health Organization. Improving maternal and newborn health and survival and reducing stillbirth - Progress report 2023. Geneva, Switzerland: World Health Organization; 2023. Available: https://www.who.int/publications/i/item/9789240073678. Accessed: 21 June 2024.

[6] World Health Organization. GHO | By category | Institutional births - Data by country. 2025. Available: https://apps.who.int/gho/data/view.main.SRHIBv. Accessed: 24 March 2025.

[7] Stevenson AG Tooke L Edwards EM Mangiza M Horn D Heys M The use of data in resource limited settings to improve quality of care. Semin Fetal Neonatal Med. 2021;26:101204. 10.1016/j.siny.2021.101204 33579628

[8] World Health Organization. Monitoring the building blocks of health systems: a handbook of indicators and their measurement strategies. Geneva, Switzerland: World Health Organization; 2010. Available: https://iris.who.int/handle/10665/258734. Accessed: 21 June 2024.

[9] Nicol E Bradshaw D Uwimana-Nicol J Dudley L Perceptions about data-informed decisions: an assessment of information-use in high HIV-prevalence settings in South Africa. BMC Health Serv Res. 2017;17:765. 10.1186/s12913-017-2641-1 29219085 PMC5773892

[10] Mboera LEG Rumisha SF Mbata D Mremi IR Lyimo EP Joachim C Data utilisation and factors influencing the performance of the health management information system in Tanzania. BMC Health Serv Res. 2021;21:498. 10.1186/s12913-021-06559-1 34030696 PMC8146252

[11] Nemser B Data-informed decision-making for life-saving commodities investments in Malawi: A qualitative case study. Malawi Med J. 2018;30:111. 10.4314/mmj.v30i2.11 30627339 PMC6307067

[12] Seid MA Bayou NB Ayele FY Zerga AA Utilization of Routine Health Information from Health Management Information System and Associated Factors Among Health Workers at Health Centers in Oromia Special Zone, Ethiopia: A Multilevel Analysis. Risk Manag Healthc Policy. 2021;14:1189–98. 10.2147/RMHP.S285604 33776496 PMC7987307

[13] Abdisa AB Hajito KW Daka DW Ergiba MS Senay AB Abdi KL Health workers’ use of routine health information and related factors at public health institutions in Illubabor Zone, Western Ethiopia. BMC Med Inform Decis Mak. 2022;22:140. 10.1186/s12911-022-01881-y 35610716 PMC9131521

[14] Chanyalew MA Yitayal M Atnafu A Tilahun B Assessment of data demand for informed-decisions among health facility and department heads in public health facilities of Amhara Region, northwest Ethiopia. Health Res Policy Syst. 2023;21:62. 10.1186/s12961-023-01006-5 37365611 PMC10291749

[15] Lemma S Janson A Persson LÅ Wickremasinghe D Källestål C Improving quality and use of routine health information system data in low- and middle-income countries: A scoping review. PLoS ONE. 2020;15:e0239683. 10.1371/journal.pone.0239683 33031406 PMC7544093

[16] Rendell N Lokuge K Rosewell A Field E Factors That Influence Data Use to Improve Health Service Delivery in Low- and Middle-Income Countries. Glob Health Sci Pract. 2020;8:566–81. 10.9745/GHSP-D-19-00388 33008864 PMC7541116

[17] Tilahun B Derseh L Atinafu A Mamuye A Mariam TH Mohammed M Leveland contributing factors of health data quality and information use in two districts in Northwest Ethiopia: social-ecological perspective. BMC Med Inform Decis Mak. 2021;21:373. 10.1186/s12911-021-01741-1 34972511 PMC8719401

[18] MEASURE Evaluation. PRISM: Performance of Routine Information System Management Series. 2024. Available: https://www.measureevaluation.org/prism.html. Accessed: 8 October 2024.

[19] Ayele M Mariani I Abathun F Mouhamadou O Minja J Kananura RM Functionalities of the electronic RHIS related to newborn data: findings of the IMPULSE study across in Uganda, Ethiopia, Tanzania and Central African Republic. J Glob Health. 2025. 10.7189/jogh.15.04330 PMC12677248 41343171

[20] Kananura RM Dalena P Cora LG Abathun F Mouhamadou O Minja J Availability and the quality of essential newborn health data within routine health facility data: findings of the IMPULSE observational study in Uganda, Ethiopia, Tanzania and Central African Republic. J Glob Health. 2025. 10.7189/jogh.15.04359 PMC12677238 41343194

[21] Mouhamadou O Cora LG Minja J Abathun F Kananura RM Ayele M Users’ capabilities related to the electronic RHIS for newborn and stillbirth indicators: quantitative and qualitative findings of the IMPULSE study across 151 sites in Central African Republic, Ethiopia, Tanzania and Uganda. J Glob Health. 2025. 10.7189/jogh.15.04239 PMC12634022 41264542

[22] Mariani I Abathun F Mouhamadou O Minja J Kananura RM Tognon F Organizational and management factors and related end-users’ perspectives relevant to newborn and stillbirth data at different levels of the health system: findings of the IMPULSE study in Uganda, Ethiopia, Tanzania and Central African Republic. J Glob Health. 2025. 10.7189/jogh.15.04329 PMC12677244 41343156

[23] von Elm E Altman DG Egger M Pocock SJ Gøtzsche PC Vandenbroucke JP The Strengthening the Reporting of Observational Studies in Epidemiology (STROBE) statement: guidelines for reporting observational studies. J Clin Epidemiol. 2008;61:344–9. 10.1186/s12911-017-0509-2 18313558

[24] The London School of Hygiene & Tropical Medicine UK, Ifakara Health Institute Tanzania, International Centre for Diarrheal Disease Research Bangladesh, Data for Impact. Every Newborn-Measurement Improvement for Newborn & Stillbirth Indicators EN-MINI-PRISM Tools for Routine Health Information Systems. 2023. Available: https://www.data4impactproject.org/publications/en-mini-tools-1-6/. Accessed: 12 August 2024.

[25] The Operational Research and Coaching for Analysts (ORCA)- participants & team Adane A Adege TM Ahmed MM Anteneh HA Ayalew ES Exploring data quality and use of the routine health information system in Ethiopia: a mixed-methods study. BMJ Open. 2021;11:e050356. 10.1136/bmjopen-2021-050356 34949613 PMC8710857

[26] Shiferaw AM Zegeye DT Assefa S Yenit MK Routine health information system utilization and factors associated thereof among health workers at government health institutions in East Gojjam Zone, Northwest Ethiopia. BMC Med Inform Decis Mak. 2017;17:116. 10.1136/bmjopen-2021-050356 28784115 PMC5545835

[27] Dagnew E Woreta SA Shiferaw AM Routine health information utilization and associated factors among health care professionals working at public health institution in North Gondar, Northwest Ethiopia. BMC Health Serv Res. 2018;18:685. 10.1186/s12913-018-3498-7 30180897 PMC6122568

[28] Kuehne A Roberts L Learning from health information challenges in the Central African Republic: where documenting health and humanitarian needs requires fresh approaches. Confl Health. 2021;15:68. 10.1186/s13031-021-00405-1 34530880 PMC8444360

[29] Hoxha K Hung YW Irwin BR Grépin KA Understanding the challenges associated with the use of data from routine health information systems in low- and middle-income countries: A systematic review. Health Inf Manag. 2022;51:135–48. 10.1177/1833358320928729 32602368

[30] Molenaar J Beňová L Christou A Lange IL van Olmen J Travelling numbers and broken loops: A qualitative systematic review on collecting and reporting maternal and neonatal health data in low-and lower-middle income countries. SSM Popul Health. 2024;26:101668. 10.1016/j.ssmph.2024.101668 38645668 PMC11031824

[31] Abera A Tolera A Tusa BS Weldesenbet AB Tola A Shiferaw T Experiences, barriers, and facilitators of health data use among performance monitoring teams (PMT) of health facilities in Eastern Ethiopia: A qualitative study. PLoS One. 2023;18:e0285662. 10.1371/journal.pone.0285662 37167309 PMC10174501

[32] Tilahun H Abate B Belay H Gebeyehu A Ahmed M Simanesew A Drivers and Barriers to Improved Data Quality and Data-Use Practices: An Interpretative Qualitative Study in Addis Ababa, Ethiopia. Glob Health Sci Pract. 2022;10 Supplement 1:e2100689. 10.9745/GHSP-D-21-00689 36109055 PMC9476478

[33] Braa J Heywood A Sahay S Improving quality and use of data through data-use workshops: Zanzibar, United Republic of Tanzania. Bull World Health Organ. 2012;90:379–84. 10.2471/BLT.11.099580 22589572 PMC3341693

[34] Mutale W Chintu N Amoroso C Awoonor-Williams K Phillips J Baynes C Population Health Implementation and Training – Africa Health Initiative Data Collaborative. Improving health information systems for decision making across five sub-Saharan African countries: Implementation strategies from the African Health Initiative. BMC Health Serv Res. 2013;13:S9. 10.1186/1472-6963-13-S2-S9 23819699 PMC3668230

[35] Ouedraogo M Kurji J Abebe L Labonté R Morankar S Bedru KH A quality assessment of Health Management Information System (HMIS) data for maternal and child health in Jimma Zone, Ethiopia. PLoS One. 2019;14:e0213600. 10.1371/journal.pone.0213600 30856239 PMC6411115

