# Supplementary material for: Facility newborn and stillbirth data use and enabling factors at different levels of the health system: findings of the IMPULSE study across 142 sites in the Central African Republic, Ethiopia, Tanzania, and Uganda
Source: J Glob Health. 2025 Dec 19;15:04295. doi: 10.7189/jogh.15.04295 (PMC12715746; doi:10.7189/jogh.15.04295)
Supplement: Online Supplementary Document [file jogh-15-04295-s001.pdf]

Supplement to: Abathun F, Dalena P, Kananura RM, Minja J, Mouhamadou O, Ayele M, Day L, Tognon F, Cora LG, Mariani I, Geremia S, Putoto G, Lawn EJ, Awel T, Bundala F, Shamba D, Waiswa P, Lazzerini M. Facility newborn and stillbirth data use and enabling factors at different levels of the health system: findings of the IMPULSE study across 142 sites in the Central African Republic, Ethiopia, Tanzania, and Uganda. J Glob Health. 2025;15:04295.

Online supplementary file

TABLES

Table S1. The Strengthening the Reporting of Observational Studies (STROBE) Checklist 1
Table S2. Characteristics of the regions 3
Table S3. Sampling criteria 4
Table S4. Sample characteristics by IMPULSE administrative unit and regions 5
Table S5. Data processing and use at the facility level 7
Table S6. Data processing and use at the data office level 9
Table S7. Average scores for data processing and use 11
Table S8. Data dissemination to the general public 12
Table S9. Enabling factors at the facility level 13
Table S10. Enabling factors at the data office level 14
Table S11. End users' perspective 15

FIGURES

Figure S1. PRISM framework 16
Figure S2. Geographical distribution of the regions included in the IMPULSE study 17
Figure S3. IMPULSE study: End users' perspective on RHIS improvement (N = 92 health facilities, N = 49 subnational offices, N = 141 respondents) 18

TABLES

Table S1. The Strengthening the Reporting of Observational Studies (STROBE) Checklist

	Item No.	Recommendation	Pages
**Title and abstract**	1	(*a*) Indicate the study's design with a commonly used term in the title or the abstract	1-3
		(*b*) Provide in the abstract an informative and balanced summary of what was done and what was found	3
**Introduction**			
Background/rationale	2	Explain the scientific background and rationale for the investigation being reported	4-5
Objectives	3	State specific objectives, including any prespecified hypotheses	5
**Methods**			
Study design	4	Present key elements of study design early in the paper	5
Setting	5	Describe the setting, locations, and relevant dates, including periods of recruitment, exposure, follow-up, and data collection	5
Participants	6	(*a*) Give the eligibility criteria, and the sources and methods of selection of participants	5
Variables	7	Clearly define all outcomes, exposures, predictors, potential confounders, and effect modifiers. Give diagnostic criteria, if applicable	6
Data sources/ measurement	8*	For each variable of interest, give sources of data and details of methods of assessment (measurement). Describe comparability of assessment methods if there is more than one group	6-7
Bias	9	Describe any efforts to address potential sources of bias	6-8
Study size	10	Explain how the study size was arrived at	6
Quantitative variables	11	Explain how quantitative variables were handled in the analyses. If applicable, describe which groupings were chosen and why	7
Statistical methods	12	(*a*) Describe all statistical methods, including those used to control for confounding	7
		(*b*) Describe any methods used to examine subgroups and interactions	7
		(*c*) Explain how missing data were addressed	7
		(*d*) If applicable, describe analytical methods taking account of sampling strategy	N/A
		(*e*) Describe any sensitivity analyses	-
**Results**			
Participants	13*	(a) Report numbers of individuals at each stage of study—e.g. numbers potentially eligible, examined for eligibility, confirmed eligible, included in the study, completing follow-up, and analysed	8
		(b) Give reasons for non-participation at each stage	-
		(c) Consider use of a flow diagram	-
Descriptive data	14*	(a) Give characteristics of study participants (e.g. demographic, clinical, social) and information on exposures and potential confounders	8
		(b) Indicate number of participants with missing data for each variable of interest	Supplementary File
Outcome data	15*	Report numbers of outcome events or summary measures	9-13

Main results	16	(*a*) Give unadjusted estimates and, if applicable, confounder-adjusted estimates and their precision (e.g., 95% confidence interval). Make clear which confounders were adjusted for and why they were included	9-13
		(*b*) Report category boundaries when continuous variables were categorized	-
		(*c*) If relevant, consider translating estimates of relative risk into absolute risk for a meaningful time period	-
Other analyses	17	Report other analyses done—eg analyses of subgroups and interactions, and sensitivity analyses	-
**Discussion**			
Key results	18	Summarise key results with reference to study objectives	13-16
Limitations	19	Discuss limitations of the study, taking into account sources of potential bias or imprecision. Discuss both direction and magnitude of any potential bias	16
Interpretation	20	Give a cautious overall interpretation of results considering objectives, limitations, multiplicity of analyses, results from similar studies, and other relevant evidence	14
Generalisability	21	Discuss the generalisability (external validity) of the study results	16
**Other information**			
Funding	22	Give the source of funding and the role of the funders for the present study and, if applicable, for the original study on which the present article is based	16

*Give information separately for exposed and unexposed groups

N/A – not applicable

Table S2 Characteristics of the regions

Key characteristics	CAR					Ethiopia					Tanzania					Uganda					Overall
	Bangui C.A.	Health region 1	Health region 2	Health region 7	Total	Addis Ababa C.A.	Oromia	Amhara/Gambella	South Ethiopia/ Sidama	Total	Dar es Salaam C.A.	Iringa	Shinyanga	Simiyu	Total	Kampala C.A.	Lango	Karamoja	West-Nile	Total	
Setting																					
Urban	Yes	-	-	-	1	Yes	-	-	-	1	Yes	-	-	-	1	Yes	-	-	-	1	4
Rural	-	-	Yes	-	1	-	-	-	-	2	-	-	Yes	Yes	2	-	-	Yes	-	1	6
Mixed	-	Yes	-	Yes	2	-	Yes	Yes	Yes	1	-	Yes	-	-	1	-	Yes	-	Yes	2	6
Hard to reach /disadvantaged area	-	-	-	-	-	-	-	-	Yes, semi nomadic population, flood risk	1	-	-	Yes	Yes	2	-	-	Yes, semi nomadic population	-	1	4
Humanitarian setting	Yes, conflict	Yes, conflict	Yes, conflict	Yes, conflict	4	-	-	Yes, Gambella refugees	-	1	-	-	-	-	-	-	-	-	Yes, refugees	1	6

C.A. – City Administration; CAR - Central African Republic

Table S3. Sampling criteria

**Facility type**	**Criteria**
Health facilities level	
In each country	
3rd level of referral (National)	1
In each Region	
3rd level of referral (Regional)	1
2nd level of referral (Subnational / District) Public	2
2nd level of referral (Subnational / District) Not for Profit	1 (if existing, and allowing)
2nd level of referral (Subnational / District) Private	1 (if existing, and allowing)
1st level of referral (Primary Hospital / Health Centre with CEmONC) - exept CAR BEmONC Public	3
1st level of referral (Primary Hospital / Health Centre with CEmONC) - exept CAR BEmONC Not for Profit	1-2 (if existing, and allowing)
1st level of referral (Primary Hospital / Health Centre with CEmONC) - exept CAR BEmONC Private	1-2 (if existing, and allowing)
Data offices level	
District /Subnational health office	All data offices related to selected facilities
Regional health office	Yes
National data office	Yes

BEmONC - basic emergency obstetric and neonatal care; CAR – Central African Republic; CEmONC - comprehensive emergency obstetric and newborn care

Table S4. Sample characteristics by IMPULSE administrative unit and regions for tool 2a ad 2b

Tool utilised	N indicators utilised	Sample characteristics		Overall		CAR		Ethiopia		Tanzania		Uganda	
				N	%	N	%	N	%	N	%	N	%
Tool 2a	10	Offices characteristics		N = 49		N = 6		N = 6		N = 17		N = 20	
			District health data office	46	93.9	6	100	6	100	14	82.4	20	100
			Regional health data office	3	6.1	0	0	0	0	3	17.6	0	0
		Regions	CAR	6	12.2								
			Health region 1	1	2.0	1	16.7	-	-	-	-	-	-
			Health region 2	2	4.1	2	33.3	-	-	-	-	-	-
			Health region 7	3	6.1	3	50	-	-	-	-	-	-
			Bangui City Administration	0	0	0	0	-	-	-	-	-	-
			Ethiopia	6	12.2								
			Addis Ababa City Administration	0	0	-	-	0	0	-	-	-	-
			Oromia	2	4.1	-	-	2	33.3	-	-	-	-
			Amhara and Gambella	1	2	-	-	1	16.7	-	-	-	-
			South Ethiopia and Sidama	3	6.1	-	-	3	50	-	-	-	-
			Tanzania	17	34.7								
			Iringa	6	12.2	-	-	-	-	6	35.3	-	-
			Shinyanga	5	10.2	-	-	-	-	5	29.4	-	-
			Simiyu	6	12.2	-	-	-	-	6	35.3	-	-
			Uganda	20	40.8								
			Lango	6	12.2	-	-	-	-	-	-	6	30
			Karamoja	8	16.3	-	-	-	-	-	-	8	40
			West-Nile	6	12.2	-	-	-	-	-	-	6	30
			Kampala City Administration	0	0	-	-	-	-	-	-	0	0
Tool 2b	19	Health facilities characteristics		N = 93		N = 14		N = 24		N = 27		N = 28	
			Third level of referral (national/regional) hospital	15	16.2	4	28.6	3	12.5	3	11.1	5	17.9
			Second level of referral hospital	39	41.9	3	21.4	10	41.7	15	55.6	11	39.3
			First level of referral health facility	39	41.9	7	50	11	45.8	9	33.3	12	42.9
		Regions	CAR	14	14.7								
			Health region 1	3	3.2	3	21.4	-	-	-	-	-	-
			Health region 2	4	4.2	4	28.6	-	-	-	-	-	-
			Health region 7	4	4.2	4	28.6	-	-	-	-	-	-
			Bangui City Administration	3	3.2	3	21.4	-	-	-	-	-	-
			Ethiopia	24	25.3								
			Addis Ababa City Administration	3	3.2	-	-	3	12.5	-	-	-	-
			Oromia	9	9.5	-	-	9	37.5	-	-	-	-
			Amhara and Gambella	3	3.2	-	-	3	12.5	-	-	-	-
			South Ethiopia and Sidama	9	9.5	-	-	9	37.5	-	-	-	-
			Tanzania	27	28.4								
			Dar es Salaam City Administration	2	2.1	-	-	-	-	2	6.9	-	-
			Iringa	10	10.5	-	-	-	-	10	34.5	-	-
			Shinyanga	9	9.5	-	-	-	-	9	31.0	-	-
			Simiyu	8	8.4	-	-	-	-	8	27.6	-	-
			Uganda	28	29.5								
			Lango	9	9.5	-	-	-	-	-	-	9	32.1
			Karamoja	7	7.4	-	-	-	-	-	-	7	25

		West-Nile	11	11.6	-	-	-	-	-	-	11	39.3
		Kampala City Administration	1	1.1	-	-	-	-	-	-	1	3.6
	Other characteristics	Urban/rural										
		Urban	61	65.6	13	92.4	18	75	21	72.4	13	43.3
		Rural	32	34.4	1	7.1	6	25	8	27.6	17	56.7
		Managing authority										
		Government/public	71	76.3	12	85.8	18	75	22	75.8	23	76.7
		Private for profit	9	9.7	1	7.1	5	20.8	3	10.3	0	0
		Private not-for-profit	13	14.0	1	7.1	1	4.2	4	13.9	7	23.3

CAR - Central African Republic

Table S5. Data processing and use at the facility level

	CAR<br>N =14	Ethiopia<br>N = 24	Tanzania<br>N = 27	Uganda<br>N = 28	Overall<br>N = 93
**Data processing**			Percentage		
Facility prepares data visual showing achievement towards targets	7.1	75	51.9	92.9	63.5
Data visual on:					
Maternal health care	0	100	85.7	96.2	79.6
Neonatal and child healthcare	0	94.4	57.1	88.5	67.6
Top causes of neonatal morbidity and mortality	100	22.2	42.9	88.5	59.9
Other	0	11.1	0	26.9	10.9
Facility has access to analysed RHIS data (tables, charts)	21.4	91.7	55.6	89.3	69.9
Report produced based on an analysis of RHIS data	28.6	66.7	63	67.9	60.2
Up to date reports available in the facility displaying:					
Summary RHIS reports newborn and stillbirth data (past 3 months)	28.6	83.3	63	78.6	67.8
Demographic data for calculating coverage	0	50	22.2	60.7	37.6
Indicators for impact calculated (last 3 months)	14.3	62.5	48.1	57.1	49.4
Comparison facility vs district/national targets	0	41.7	18.5	35.7	26.9
Comparison of data over time	7.1	66.7	37	57.1	46.2
Comparison of sex-disaggregated data	7.1	12.5	18.5	25	17.2
Comparison of service coverage	0	33.3	11.1	35.7	22.6
**Data use for quality improvement**					
Reports contains recommendation on:					
Service coverage	0	81.2	41.2	78.9	56.7
Hospital/health centre performance	0	62.5	58.8	84.2	58.5
Top causes of neonatal mortality/morbidity	25	18.8	76.5	73.7	53
Identification of emerging issues/epidemics	50	37.5	11.8	63.2	39.7
Medicine stock outs	25	62.5	41.2	63.2	50.9
Human resource management	0	75	35.3	68.4	50.2
Sex disaggregated data	50	6.2	23.5	47.4	30.2
Routine team meetings for performance monitoring management	14.3	83.3	81.5	85.7	73.1
Minutes of performance maintained (last 3 months)	0	35	45.5	54.2	38.6
The following topics were discussed in the performance monitoring/ management meetings:					
RHIS management, such as newborn/ stillbirth data quality, completeness, or timeliness of reporting	0	71.4	80	92.3	69.4
If *yes*, any decisions based on the discussions on newborn/ stillbirth RHIS-related issues	0	80	87.5	66.6	66.1
If *yes*, has any follow-up action taken place on the decisions	0	75	100	75	70.9
Were discussions held to review key performance targets, such as:					
Service coverage	0	71.4	40	46.1	43.9
Hospital/health centre performance	0	71.4	80	53.8	57.8
Top causes of neonatal mortality/morbidity	0	57.1	60	53.8	48.3
Identification of emerging issues/epidemics	0	42.8	40	38.4	34.2
Medicine stocks out	0	71.4	40	46.1	43.9
Human resource management	0	71.4	70	46.1	52.6
Sex disaggregated data	0	14.3	10	46.1	20.5
Any decision made based on data discussion on:					
Formulation of plans	0	66.7	77.8	80	63.9
Budget preparation	0	33.3	11.1	70	32.9
Budget reallocation	0	33.3	22.2	70	36.1
Medicine supply and drug management	0	33.3	66.7	60	46

Human resource management	0	83.3	77.8	60	62.1
Advocacy	0	66.7	11.1	50	35.5
Promotion of service quality/improvement	0	83.3	77.8	60	62.1
Reducing the gender gap	0	16.7	0	40	16.3
No action required at this time for newborn care and preventing stillbirths	0	16.7	0	40	16.3
Minutes performance monitoring/ management meetings circulated to all	0	100	33.3	72.7	57.4
**Frequency of PMT meetings**					
Expected frequency of PMT meetings:					
Weekly	0	0	22.7	20.8	12.8
Monthly	100	95	50	50	69.1
Weekly	0	5	27.3	29.2	18
Biannually	0	0	0	0	0
Annually	0	0	0	0	0
No schedule	0	0	0	0	0
How many times did the PMT meetings take place during the past 3 months:					
More than four times	0	0	22.8	29.2	15.4
Four times	0	5	4.6	0	2.6
Three times	100	50	45.4	29.2	49.9
Two times	0	25	9	0	9.1
One time	0	20	18.2	41.6	22.9
Not once	0	0	0	0	0
**Number of key indicators ≥ 80%**	1	7	1	7	0

Key indicators are highlighted in grey

CAR – Central African Republic; PMT – performance monitoring management; RHIS – routine health information system

Table S6. Data processing and use at the data office level

	CAR N = 6	Ethiopia N = 6	Tanzania N = 17	Uganda N = 20	Overall N = 49
**Data processing**			Percentage		
District office prepares data visual showing achievement towards targets	50	100	58.8	95	77.5
Data visual on:					
Maternal health care	66.7	100	90	100	92.4
Neonatal and child healthcare	66.7	100	90	94.7	90.3
Top causes of neonatal morbidity and mortality	0	16.7	80	68.4	57.7
Other	33.3	0	10	42.1	24.7
District has access to analysed RHIS data (tables, charts)	0	33.3	0	5	6.1
Report produced based on an analysis of RHIS data	16.7	100	82.4	80	75.5
Up to date bulletin available at DHO displaying:					
Summary RHIS reports newborn and stillbirth data (past 3 months)	16.7	83.3	82.4	90	77.6
Demographic data for calculating coverage	0	66.7	64.7	85	65.3
Indicator for impact calculated (last 3 months)	16.7	50	70.6	85	67.3
Comparison of impact indicators among health facilities	16.7	66.7	58.8	75	61.2
Comparison vs district/national targets	16.7	83.3	64.7	70	63.3
Comparison of data over time	16.7	66.7	70.6	85	69.4
Comparison of sex disaggregated data	0	16.7	35.3	45	32.7
Comparison of service coverage	16.7	66.7	41.2	55	46.9
**Data use for quality improvement**					
Reports contains recommendation on:					
Service coverage	0	100	47.1	70	57.2
Hospital/health centre performance	16.7	100	58.8	75	65.3
Top causes of neonatal mortality/morbidity	0	16.7	70.6	70	55.1
Identification of emerging issues/epidemics	16.7	66.7	29.4	65	46.9
Medicine stock outs	0	66.7	64.7	70	59.2
Human resource management	0	83.3	41.2	70	53.1
Sex disaggregated data	0	16.7	23.5	60	34.7
Routine team meetings for performance monitoring management (PMT)	50	83.3	100	95	89.8
Minutes of PMT meetings maintained (last 3 months)	33.3	40	52.9	57.8	50.9
PMT record containing:					
Discussion on newborn and still birth data quality	0	33.3	52.9	45	40.8
Decisions made on newborn and stillbirth data quality	0	33.3	47.1	40	36.7
Follow up action taken	0	16.7	41.2	35	30.6
Any decision made based on data discussion:					
Formulation of plans	0	33.3	47.1	45	38.8
Budget preparation	0	16.7	29.4	45	30.6
Budget reallocation	0	33.3	23.5	50	32.6
Medicine supply and drug management	0	33.3	47.1	45	38.8
Human resource management	0	33.3	41.2	45	36.7
Advocacy	0	33.3	23.5	35	26.5
Planning health services	0	33.3	41.2	40	34.7
Promotion of service quality/improvement	0	33.3	47.1	40	36.7
Reducing the gender gap	0	0	0	35	14.3
Involvement of the community and local government	0	33.3	29.4	45	32.6
No action required at this time	0	0	5.9	15	8.2
Head of the district office attended any meeting	16.7	33.3	41.2	50	40.8

Frequency and content of PMT meetings					
Expected frequency of PMT meetings:					
Weekly	0	0	0	0	0
Monthly	0	100	47	15.8	35
Quarterly	66.7	0	53	84.2	60.9
Biannually	0	0	0	0	0.0
Annually	0	0	0	0	0.0
No schedule	33.3	0	0	0	4.1
Actual frequency of PMT meetings (past 3 months):					
More than four times	0	0	11.7	5.2	6.2
Four times	0	0	0	5.2	2.1
Three times	0	20	23.6	10.6	14.9
Two times	0	0	0	5.2	2.1
One time	66.7	60	52.9	58	57.5
Not once	33.3	20	11.8	15.8	17.1
**Number of key indicators ≥ 80%**	0	8	4	7	1

Key indicators are highlighted in grey

CAR – Central African Republic; DHO – district health office; RHIS - routine health information system; PMT - performance monitoring management

Table S7. Average scores (composite PRISM indexes) for data processing and use

**Facility level**	**Overall**	**CAR**	**Ethiopia**	**Tanzania**	**Uganda**
	Percentage				
Average score on the use of routine data for RHIS quality improvement, performance review, and evidence-based decision making	22.8	0	19.2	28.9	31.4
Average score on the use of routine data for RHIS quality improvement, performance review, and evidence-based decision making (among facilities maintaining performance monitoring/management meeting minutes for the three review months)	70.8	0	65.7	78	67.9
**Data office level**	Percentage				
Average score on the use of routine data for RHIS quality improvement, performance review, and evidence-based decision making	38	0	30	47.1	44.4
Average score on the use of routine data for RHIS quality improvement, performance review, and evidence-based decision making (among data offices and facilities maintaining performance monitoring/management meeting minutes for the three review months)	80.8	0	90	88.9	80

The scores are calculated following the PRISM User kit [18]
CAR – Central African Republic; RHIS – routine health information system

Table S8. Data dissemination to the general public

	CAR<br>N =14	Ethiopia<br>N = 24	Tanzania<br>N = 27	Uganda<br>N = 28	Overall<br>N = 93
**Facility level**			Percentage		
Must submit performance reports to public representative	0	45.8	48.1	50	40.8
Presented reports to public representatives (observed)	0	100	84.6	35.7	71.9
The report used data to assess health sector's progress (observed)	0	100	100	100	100
Updated website for public to access RHIS data (observed)	0	20.8	0	10.7	8.6
Performance data shared via bulletin, chalkboards and publications (observed)	0	66.7	0	35.7	27.9
**Data office level**	N = 6	N = 6	N = 17	N = 20	N = 49
Must submit performance reports to district administration/councils	0	83.3	64.7	95	71.4
Presented performance reports to district/ local administration (observed)	0	100	100	94.7	97.7
The report used data to assess health sector's progress (observed)	0	100	100	94.7	97.7
Updated website for public to access RHIS data (observed)	0	0	5.9	25	12.2
Performance data shared via bulletin, chalkboards and publications (observed)	16.7	83.3	0	45	30.6
**Number of indicators ≥ 80%**	0	6	4	4	3

Key indicators are highlighted in grey

CAR – Central African Republic; RHIS - routine health information system

Table S9. Enabling factors at the facility level

	CAR N =14	Ethiopia N = 24	Tanzania N = 27	Uganda N = 28	Overall N = 93
**Electronic systems**			Percentage		
Availability of electronic system for data entering and analysing	21.4	87.5	96.3	82.1	78.5
Data entry:					
National open-source data processing system (e.g., DHIS 2)	33.3	100	100	100	97.3
National proprietary software	0	0	0	13	4.1
Excel-based spreadsheet	100	14.3	0	56.5	26
Access-based data processing	0.0	4.8	0	13	5.5
Data analysis:					
National open-source data processing system (e.g., DHIS 2)	33.3	90.5	92.3	100	91.8
National proprietary software	0	0	0	13	95.9
Excel-based spreadsheet	100	71.4	0	56.5	42.5
Access-based data processing	0	4.8	0	13	5.5
**Guidelines**					
Availability of written guidelines on data display and use	0	75	22.2	53.6	41.9
Availability of national RHIS strategic plans and facility targets	0	83.3	33.3	64.3	50.5
**Annual plans**					
Availability of facility annual plan (current year)	7.1	87.5	63	75	64.5
Facility plan uses data from RHIS to set activities & targets	7.1	100	76.5	95.2	77.7
Facility plan includes activities and target to improve:					
Service coverage	0	90.5	35.3	66.7	53.7
Hospital/health centre performance	0	95.2	70.6	85.7	70.9
Top causes of neonatal mortality/morbidity	0	33.3	76.5	66.7	50.9
Identification of emerging issues/epidemics	0	47.6	35.3	71.4	44
Medicine stock outs	0	85.7	58.8	76.2	62.1
Human resource management	0	90.5	47.1	85.7	62.8
Reducing the gender gap	0	4.8	23.5	33.3	18.1
**Feedback systems**					
Availability of any feedback report (from district office/MOH, last 3 months)	0	45.8	70.4	53.6	48.4
Related on newborn and still birth data quality	0	90.9	68.4	93.3	84.1
Related on service performance	0	90.9	78.9	93.3	87.6
Existence of performance monitoring/management team	0	91.6	92.6	89.3	77.4
**Number of key indicators ≥ 80%**	0	10	2	6	2

Key indicators are highlighted in grey

CAR – Central African Republic; DHIS – district health information software; MOH – Ministry of Health; RHIS - routine health information system

Table S10. Enabling factors at the data office level

	CAR<br>N = 6	Ethiopia<br>N = 6	Tanzania<br>N = 17	Uganda<br>N = 20	Overall<br>N = 49
**Electronic systems**			Percentage		
Availability of electronic system for data entering and analysing	83.3	100	100	95	95.9
Data entry					
National open-source data processing system (e.g., DHIS 2)	100	50	100	100	93.9
National proprietary software	60	0	29.4	36.8	32.6
Excel-based spreadsheet	60	16.7	35.3	63.2	47.4
Access-based data processing	0	0	29.4	31.6	23.1
Data analysis					
National open-source data processing system (e.g., DHIS 2)	100	100	100	100	100
National proprietary software	60	0	29.4	36.8	32.6
Excel-based spreadsheet	60	83.3	41.2	94.7	70.5
Access-based data processing	0	0	29.4	36.8	25.2
**Guidelines**					
Availability of written guidelines on data display and use	0	100	29.4	55	44.9
Availability of national RHIS strategic plans and district targets	0	100	70.6	70	65.3
**Annual plans**					
Availability of annual plan current year	33.3	100	82.4	90	81.6
Annual plan uses data from RHIS for problem identification & target setting	16.7	100	78.6	100	82.4
Annual plan includes activities and target related to improve:					
Service coverage	0	83.3	63.6	66.6	59.4
Hospital/health centre performance	0	100	90.9	94.4	82.3
Top causes of neonatal mortality/morbidity	0	16.6	90.9	88.8	69.8
Identification of emerging issues/epidemics	0	66.6	45.4	77.7	55.6
Medicine stocks out	0	100	81.8	94.4	79.2
Human resource management	0	100	81.8	88.8	76.9
Reducing the gender gap	0	0	18.2	55.5	28.9
**Feedback systems**					
Data office sent feedback reports to health facilities (last 3 months)	16.7	100	88.2	70	73.5
Related on:					
Data quality	16.7	100	100	92.9	86.9
Service performance	0	100	100	92.9	84.9
Existence of performance monitoring/management team	50	100	100	100	93.9
**Number of key indicators ≥ 80%**	1	12	10	9	6

Key indicators are highlighted in grey

CAR – Central African Republic; DHIS – district health information software; RHIS - routine health information system

Table S11. End users' perspective

End users' perspective	Level	CAR	Ethiopia	Tanzania	Uganda
Any improvement needed	Data office	100	16.7	82.4	75
Any improvement needed	Facility	100	21.7	77.8	75

RHIS - routine health information system

FIGURES

Figure S1. PRISM framework

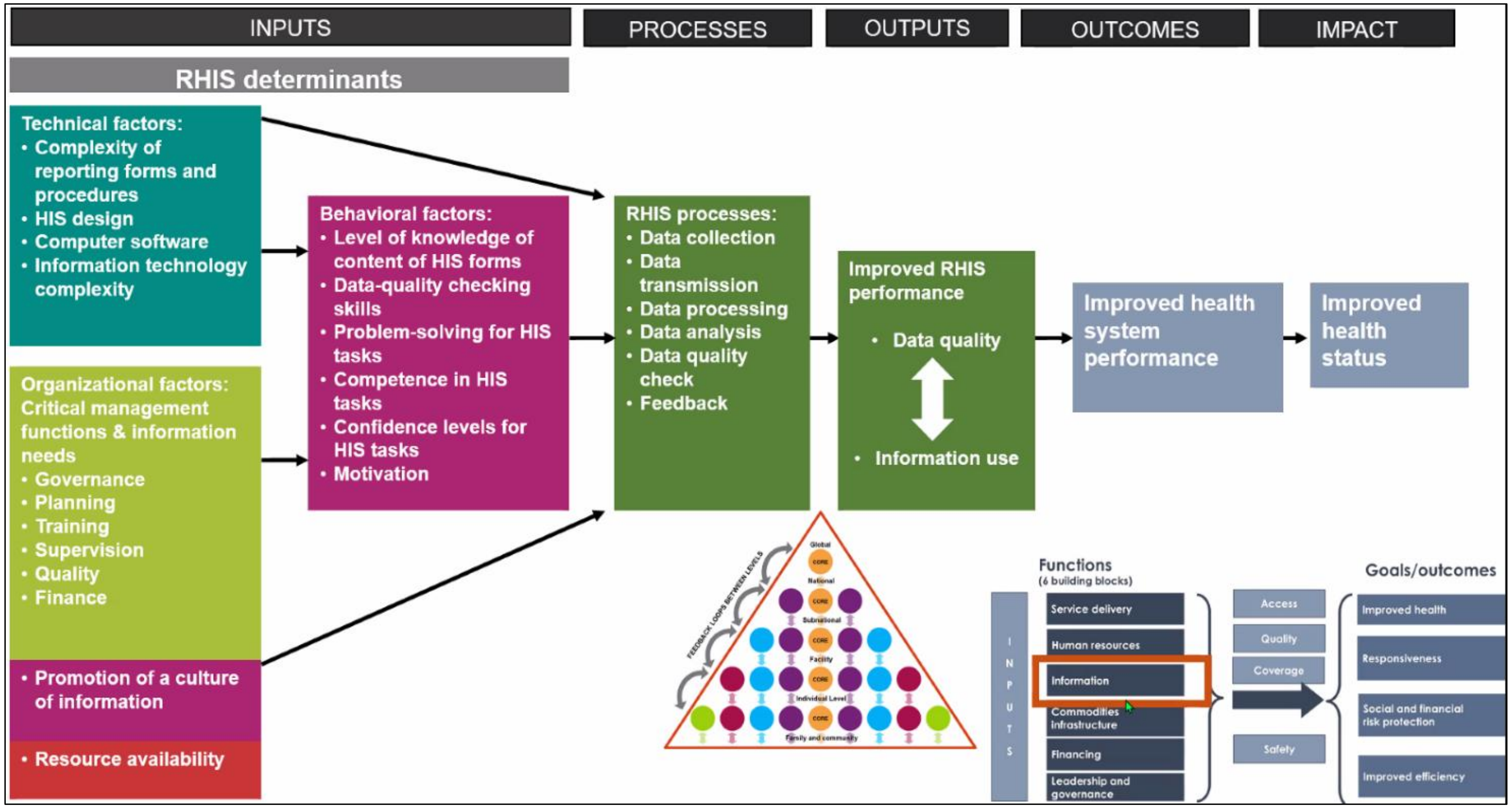

Aqil A, Lippeveld T, Hozumi D. PRISM framework: a paradigm shift for designing, strengthening and evaluating routine health information systems. Health Policy Plan. 2009;24:217–28

PRISM - performance of routine information system management; RHIS – routine health information system

Figure S2. Geographical distribution of the regions included in the IMPULSE study

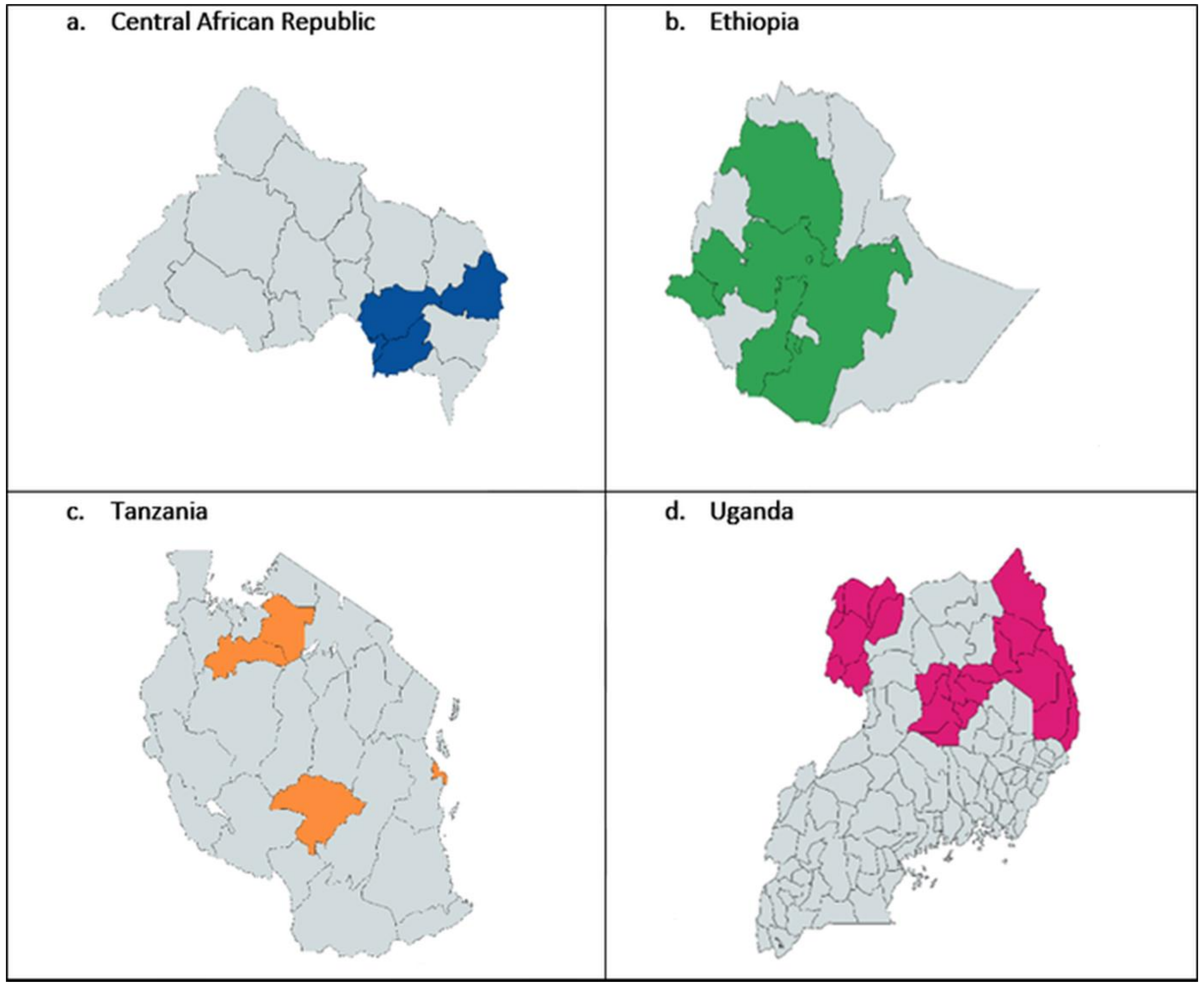

The figure shows regions included in the IMPULSE study. For Central African Republic: Bangui City Administration, Health Region 1, Health Region 2, Health Region 7; for Ethiopia: Addis Ababa City Administration, Oromia, Amhara and Gambella, South Ethiopia and Sidama; for Tanzania: Dar es Salaam City Administration, Iringa, Shinyanga, Simiyu; for Uganda: Lango, Karamoja, West-Nile, Kampala City Administration

Figure S3. IMPULSE study: End users' perspective on RHIS improvement (N = 92 health facilities, N = 49 subnational offices, N = 141 respondents)

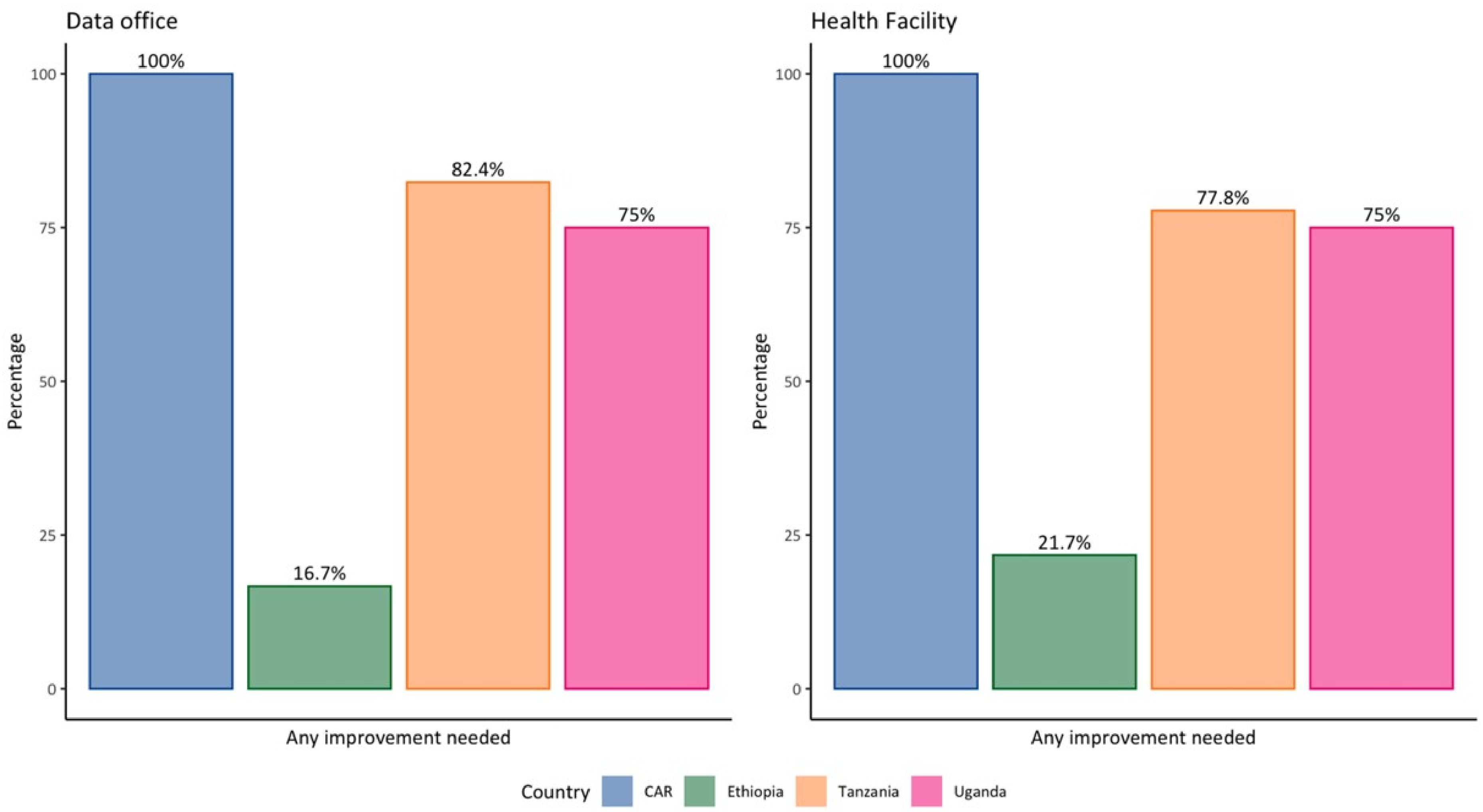

CAR – Central African Republic; RHIS – routine health information system
